# Year-round utilization of sea ice-associated carbon in Arctic ecosystems

**DOI:** 10.1038/s41467-023-37612-8

**Published:** 2023-04-07

**Authors:** Chelsea W. Koch, Thomas A. Brown, Rémi Amiraux, Carla Ruiz-Gonzalez, Maryam MacCorquodale, Gustavo A. Yunda-Guarin, Doreen Kohlbach, Lisa L. Loseto, Bruno Rosenberg, Nigel E. Hussey, Steve H. Ferguson, David J. Yurkowski

**Affiliations:** 1 Natural History Museum, London, SW7 5BD England; 2 University of Maryland Center for Environmental Science, Solomons, MD US; 3 Scottish Association for Marine Science, Oban, PA37 1QA Scotland; 4 Centre for Earth Observation Science, University of Manitoba, Winnipeg, MB Canada; 5 Québec-Océan and Takuvik, Biology Department, Laval University, Québec, QC Canada; 6 Norwegian Polar Institute, Fram Centre, Tromsø, 9296 Norway; 7 Fisheries and Oceans Canada, Freshwater Institute, Winnipeg, MB Canada; 8 Integrative Biology, University of Windsor, Windsor, ON N9B 3P4 Canada

**Keywords:** Marine biology, Biogeochemistry

## Abstract

Sea ice primary production is considered a valuable energy source for Arctic marine food webs, yet the extent remains unclear through existing methods. Here we quantify ice algal carbon signatures using unique lipid biomarkers in over 2300 samples from 155 species including invertebrates, fish, seabirds, and marine mammals collected across the Arctic shelves. Ice algal carbon signatures were present within 96% of the organisms investigated, collected year-round from January to December, suggesting continuous utilization of this resource despite its lower proportion to pelagic production. These results emphasize the importance of benthic retention of ice algal carbon that is available to consumers year-round. Finally, we suggest that shifts in the phenology, distribution and biomass of sea ice primary production anticipated with declining seasonal sea ice will disrupt sympagic-pelagic-benthic coupling and consequently the structure and the functioning of the food web which is critical for Indigenous Peoples, commercial fisheries, and global biodiversity.

## Abstract (web summary)

Koch and Brown et al. led a collaborative and comprehensive synthesis that shows the transfer of ice algal carbon is widespread throughout the Arctic marine food web and contributes to supporting organisms throughout the dark winter months

## Introduction

Arctic marine food webs are classically viewed as being supported by two ecologically distinct types of primary production, effected in temporal sequence by ice-associated (sympagic) algae in spring and (pelagic) phytoplankton in summer. Each source contributes energy to the ecosystem with varying intensity, community composition, and nutritional quality^1,2^, with phytoplankton typically far more degraded than sea ice algae when it reaches the seafloor. Additionally, the early timing of ice algal blooms and high concentrations of polyunsaturated fatty acids make this a vital resource for many primary consumers in the spring^3^. These two microalgae communities display inherent similarities resulting from their dominance by photosynthetic diatoms^4^. However, until recently, the ability to differentiate and measure these two distinct carbon sources with other methods (e.g. stable isotopes and fatty acids) was somewhat limited in certainty owing to the lack of a specific biomarker for sea ice algae.

With the steady decline of sea ice and projections of a seasonally ice-free Arctic Ocean within this century^5^, understanding how this ecosystem is responding to ongoing climate change is of critical importance. Numerous species have evolved with the seasonal extremes of the Arctic, timing their migrations, foraging, and reproduction with the phenology of ice-associated blooms^3^. The loss of seasonal sea ice will likely not only affect the primary consumers of this resource but could also drive cascading effects within the food web, including impacting coastal ecosystem resources on which Indigenous Peoples rely. The consequences to these Arctic organisms’ life-history events and their ability to adapt to an alteration in the sea-ice primary production is uncertain^6,7^.

Here, we trace source-specific highly branched isoprenoid (HBI) diatom lipid biomarkers^8,9^ to undertake the most comprehensive and quantitative spatial and temporal assessment of carbon partitioning within the Arctic marine ecosystem to date. Since certain HBI lipids are only synthesized by a minority of sea ice diatom species^10–12^, while other HBIs are produced by a similar proportion of phytoplankton species^13,14^, these proxies now make it possible to differentiate between carbon derived from these two sources in the consumers^9^. We combined HBI biomarker values from sea ice- and phytoplankton-carbon sources into a single index, termed “H-Print”^8,9^. Based on a previously established calibration^8^, we were then able to convert H-Print values into estimates of sea-ice particulate organic carbon (iPOC) directly consumed by animals^8,15^ or transferred to higher trophic organisms. So far, this approach has resulted in comparable findings of carbon sources, examples including fatty acid markers^16–18^, bulk stable isotopes^19,20^ and compound-specific stable isotope analysis of amino acids (CSIA-AA)^21,22^ and CSIA of fatty acids (CSIA- FA)^17,23,24^. Stable isotope analysis of carbon for the primary ice-associated HBI, IP_25_, has also confirmed sea ice origins^25^. These multi-proxy approaches further validate the use of iPOC values as quantitative predictors of ice-algal carbon in Arctic food webs^26^. For example, iPOC and CSIA of a source amino acid (phenylalanine) in Atlantic walrus tissues were significantly positively correlated, where high iPOC values were associated with more enriched ^13^C typical of sea-ice algae^21^. By quantifying the sea-ice algal HBI contribution (relative to phytoplankton) throughout food chains of the marine ecosystem, spanning several marginal seas and coastal areas of the Arctic, including both Pacific and Atlantic-influenced sectors, we reveal striking year-round consumption of sea ice-associated primary production. In doing so, we consider how the potential future decrease and redistribution of this carbon source will likely have dramatic consequences requiring a concerted conservation approach to mitigate this rapid biodiversity change.

## Results and discussion

### Spatial and seasonal distribution of sea ice organic carbon across organisms

**Fig. 1:**
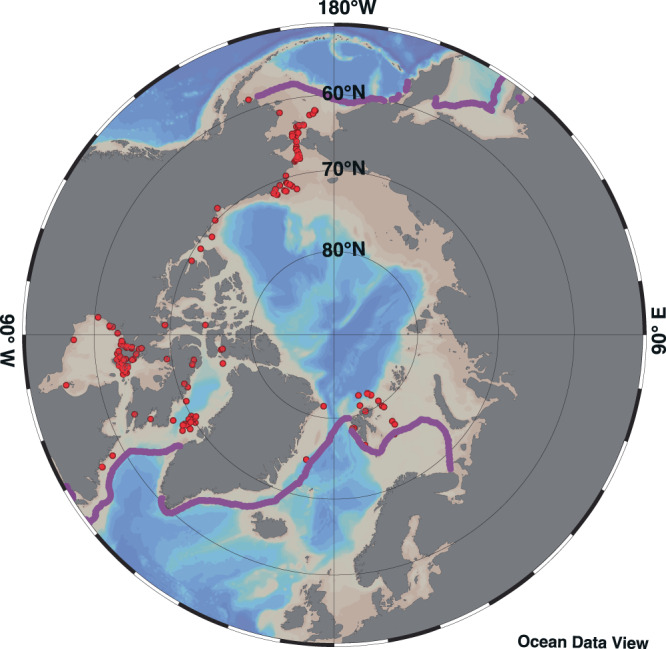
Sample collection map. Locations where animals were sampled throughout the marginal seas of the Arctic region, spanning January to December between 1982 and 2019. Magenta line represents the median March sea-ice extent (1981–2010). Map created with Ocean Data View^104^.

To reveal the importance of sea-ice organic carbon for the Arctic ecosystem, we conducted the most comprehensive synthesis to date of previously published HBI results, including additional novel biomarker data, from Arctic fauna. Our study gathers over 2300 individuals, representing 156 species, collected from approximately 60 sites across the pan-Arctic shelf ecosystems^27^ and extending from the sub- to the high-Arctic (55 − 82°N; Fig. 1). Obtained between 1982 and 2019, sampling occurred throughout the year from January to December. Owing to spatiotemporal variation in sampling in terms of numbers of species and taxonomic groups collected each year, it was not possible to examine inter-annual variability in sea ice carbon source use in relation to environmental metrics. Although this analysis does not address changes over time as a result of declining sea ice, sea-ice carbon was shown to be utilized year-round by organisms from all taxa (benthic and ice-associated invertebrates, zooplankton, fish, seabirds, and marine mammals) and habitats (benthic, sympagic or pelagic) throughout the nearly four decades of observations.

**Fig. 2:**
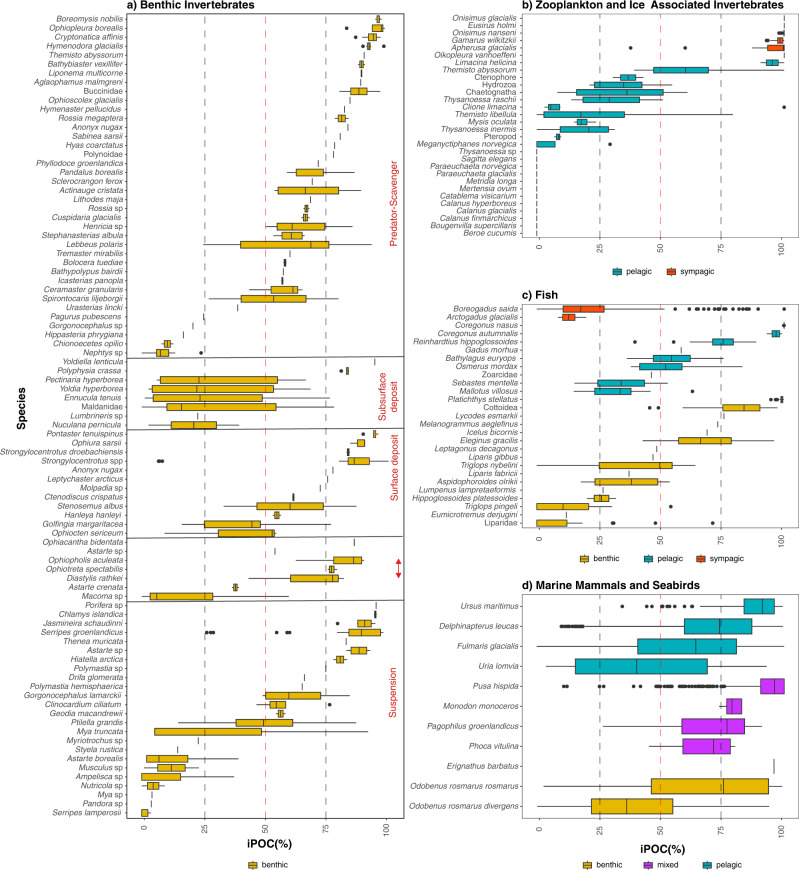
Sea ice particulate organic carbon (iPOC) content representing 159 Arctic consumers relative to phytoplankton. Species-binned HBI-based estimates of sea-ice carbon content for all individuals (*n* = 2251) sampled between 1982–2019 and grouped by major taxa including **a** benthic invertebrates sorted by feeding strategy (red text), **b** zooplankton and ice-associated invertebrates, **c** fish, and **d** seabirds and marine mammals, color-coded by habitat. 50% iPOC% (red-dashed line) is the minimum threshold to indicate that an organism recently obtained a significant proportion of their carbon from directly consuming sea ice algae or fed on organisms that rely heavily on ice algae prior to sampling. Box plots display the first to third quartiles with the vertical line representing the median values, the whiskers extend to the minimum and maximum values, and points represent outliers.

More than 96% (*n* = 2238) of organisms measured, representing 143 species, contained HBI biomarkers of both sea ice- and phytoplankton-carbon origin. In contrast, the remaining individuals (*n* = 96), from 26 species, contained only phytoplankton HBIs. Several individual zooplankton samples (small copepods, e.g. *Pseudocalanus*) collected from late summer^28^ (15 of 54 samples) and winter^29^ (17 of 43 samples) had no measurable HBI biomarkers (sea ice or phytoplankton) and were therefore excluded, as an iPOC value cannot be calculated. iPOC revealed that 67% (*n* = 1568) of animals sampled had a stronger sea-ice carbon signature than phytoplankton (Fig. 2). Where seasonal variability within samples existed, several species indicated variable reliance on both sea ice and pelagic organic carbon at different times of the year, such as the Pacific walrus (*Odobenus rosmarus divergens*), polar cod (*Boreogadus saida*) and sculpins (Cottidae). The mean iPOC values by month indicated reduced utilization of sympagic sources in May, September, and December, while the remainder of the year indicated strong sea-ice carbon utilization (>50% iPOC). Using multiple linear regressions, we tested several models to verify if iPOC values reflected an increased dependence on sea ice-organic carbon rather than an effect of sampling bias or variables relevant to prevailing sea ice conditions at time of sampling (month, year, and location collected or foraging habitat). The model inclusive of month, latitude, habitat and the interaction of month and habitat performed the best as indicated by the lowest Akaike Information Criterion (AIC) score. Habitat (*t*-value = 4.8), latitude (*t*-value = 6.24) and the interaction of month and habitat (*t*-value = −5.39) were each significant (*p* < 0.001). Month was not significant (*p* > 0.05). Given the high variability within and among species, we conducted a sensitivity analysis of this model, using habitat as the treatment. Based on the low robustness value of our sensitivity analysis (0.06, α = 0.05, see Methods) unobserved parameters would likely have a minimal impact in explaining the variability of iPOC values.

### Increased importance of springtime sea-ice carbon

**Fig. 3:**
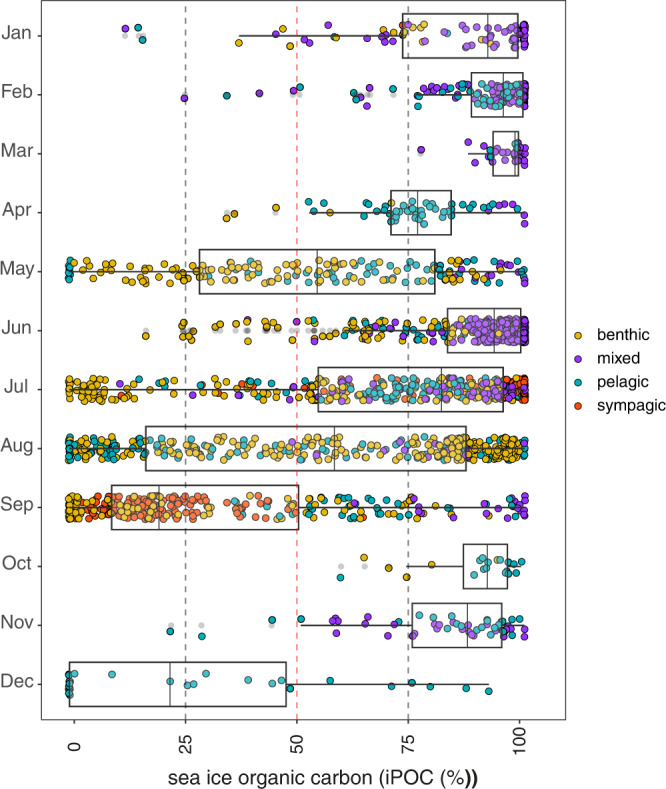
Seasonal sea ice particulate organic carbon (iPOC) content of Arctic consumers relative to phytoplankton. Monthly sampling-date binned HBI-based estimates of sea ice carbon content for all individuals with month recorded (*n* = 1940) and sampled between 1982 – 2019. Box plots display the first to third quartiles with the vertical line representing the median values and the whiskers extend to the minimum and maximum values. Data points are individual samples and are colored to represent their assigned habitat [benthic, mixed, pelagic, and sympagic].

Organisms were represented by the sympagic, benthic and pelagic components of the ecosystem and spanned all trophic levels from primary consumers including amphipods^30^, benthic invertebrates^31,32^, zooplankton^16,33^, to apex predators including polar bears^15^, seals^34^, walruses^21,35^, whales^36^, and seabirds^37^. Sea ice-derived particulate organic carbon was observed within 134 of 155 Arctic consumer species studied. There were 12 species of zooplankton (e.g., *Calanus finmarchicus, C. glacialis, C. hyperboreus*, *Metridia longa*; Fig. 2b) that had exclusively pelagic signatures at the time of sampling. Given these are the only taxa lacking in iPOC signatures entirely, which contrasts other studies^22,23,38^, this is likely the result of rapid HBI turnover rates in zooplankton and sample timing, which does not rule out the utilization of iPOC for these zooplankton species or an exclusive reliance on phytoplankton. Accordingly, we provide the first direct demonstration of the persistent role sea-ice primary production plays in supporting the energy requirements throughout a majority of the marine and coastal food webs across the Arctic, extending beyond the narrow springtime window of initial production (Fig. 3). Our results suggest a mechanism for storage and accessibility of sea-ice carbon in the Arctic environment, extending the benefits of this production beyond the spring bloom. Interestingly, ice algae contribution to the total Arctic primary production is much lower than that of phytoplankton with 28 to 211 and 355 to 1507 Tg C y^−1^ ^39,40^ respectively. In addition, both open-water and ice-bound primary production contributions are highly heterogeneous in spatial extent and most of the ice algae production occurs in the Central Arctic^41^ where the deep basin ecosystems are located^42^. Considering that the relative contribution of ice algae that are deposited on the seafloor increases with depth^32^, that the deep basins of the central Arctic are the main source of ice algae production, and that coastal areas store sea ice carbon on the seafloor, we suggest that this resource is vital to the current functioning of both deep ocean and coastal ecosystems.

Given the presumed low level of total sea ice primary production, how can sea ice-associated carbon make such a marked contribution to the ecosystem? Sea ice primary production often occurs in large concentrated biomass when the ice begins to melt, such that sinking sea ice organic matter can correspond to as much as 100% of the total carbon present in the water^43^. In these instances sea ice organic matter often exceeds the retentive capacity of pelagic consumers, where present, leading to the efficient export of sea-ice primary production to the seafloor^44–46^. This rapid carbon export results in strong sea ice-benthic coupling, especially in Arctic shelf regions where most biological activity occurs^19,47–49^. This process is often further expedited by the seasonal formation of sea ice algae aggregates of low buoyancy^50^ and/or gypsum ballasting^51,52^ that facilitate the rapid transfer of relatively fresh, undegraded organic matter to the seafloor^53^, retaining its high nutritional value^49,54^. Once the ice has retreated from the shelf, it has been demonstrated that pelagic food webs can retain almost 80% of pelagic primary production during summer^55^, largely consumed by a mass influx of marine predators migrating seasonally from temperate waters^56^ foraging on the resident pelagic and benthic primary consumers. The relatively smaller proportion of pelagic organic matter that is not retained and ultimately reaches the seafloor is often comprised of zooplankton fecal pellets and refractory organic matter that is less labile for benthic fauna when compared with sea-ice derived production^3,57,58^. It has also been suggested that bacteria may consume greater proportions of this highly degraded pelagic detritus than benthic macrofauna^22,59^.

It is commonly recognized that the contribution of ice algae to the functioning of the Arctic ecosystem is generated during the spring bloom period. Indeed, a number of organisms are observed to synchronize their life cycle with the spring bloom of the sea ice^49,54^. However, the high iPOC estimates we calculated for the majority of species throughout the year indicate that the contribution of ice algae to ecosystem function extends year-round. Furthermore, it highlights that there are still gaps in our understanding of how ice algal carbon that enters the system in the spring, and subsequently transfers throughout the ecosystem via benthic consumers^15,35,54,60,61^, will in some extent meet the energy demands of the ecosystem during the winter. In doing so, we suggest that not only is ice algal carbon important to the Arctic ecosystem throughout the year, but that its role would be even more critical in winter.

### Dynamic resource utilization following sea ice retreat

A number of species indicated utilization of both sea ice and pelagic resources particularly during the spring and summer months (Figs. 2–3). This notable shift of species using both resources follow the timing of the initial breakup of sea ice as early as May and into September. Typically, the ice algae bloom is followed by, or seeds, a phytoplankton bloom in the summer. There may also be another phytoplankton bloom in early autumn with the remineralization of nutrients and water column mixing^62^. However, the majority of samples in these months, with the exception of May and September, displayed a strong indication of sea-ice organic carbon. Declining sea ice and the subsequent redistribution or reduction of ice algal production may disproportionately impact specialized Arctic species that are either highly or partially dependent on sea-ice carbon. While some species likely exhibit dietary plasticity, there remains the potential for cascading effects throughout the food web that would drive a loss of linkages associated with specialized sea ice-carbon-dependent species^63^.

The role of sea-ice particulate organic carbon in the pelagic food web appears to have greater sensitivity to seasonal changes. Although HBI-lipid turnover rates are not determined for all species sampled, previous observations indicate turnover rates for HBI lipids of days to weeks within consumers^64–66^. Previous studies have identified the trophic transfer of HBIs within consumers and these results suggest there is no bioaccumulation in consumer tissues^15,34,36,66^. Based on the short depuration period, we suggest that pelagic primary consumers, will have HBI levels consistent with the most recent bloom (ice algae or phytoplankton). As indicated by our analysis, season cannot fully account for the low sea-ice carbon values observed but can very likely explain low to moderate values observed in species such as the ice or arctic cod (*Arctogadus glacialis*), polar cod, and *Calanus* copepods. A low transfer of HBIs in the copepod-associated pelagic food web could also explain the surprisingly low iPOC values in polar cod and ice cod compared to other fishes (Fig. 2c). We were unable to include seasonal changes in sea-ice organic carbon in any one particular species, but based on several ecologically important species representing the pelagic food web in this study, zooplankton (e.g. copepods, amphipods) from the Barents Sea in the late summer^28^ and winter of 2019^29^, had the lowest sea-ice carbon signatures at the time of sampling. There has also been evidence of low importance of ice algal carbon for some ice-associated amphipods (*Apherusa glacialis*) in the spring in the Barents Sea^33^, which contrasts with observations from the Central Arctic Ocean^33,38^ and those presented here from the Nansen Basin in July (Fig. 2b). These Barents Sea studies concluded that the observed pelagic and ice-associated species did not rely primarily on sea-ice algae, but rather used this resource opportunistically to supplement their diet at certain times of the year. With short turnover rates of HBIs and without recent ice algal production, the likelihood of observing ice-associated HBIs in late summer through winter in pelagic and ice-associated primary consumers is perhaps not surprising. There may also be higher relative importance of pelagic food sources on a regional basis, such as the Barents Sea^29,33^. Arctic pelagic species are well adapted to the seasonality of varying food sources, but how they respond to the projected shifts in primary production remains uncertain^33^. For a broader global comparison, ice algal carbon was found to be an important resource for the pelagic food web in the Southern Ocean during the winter^67^ and likely has similar importance in both polar regions.

There may also be population-level dynamics in relation to sea ice observed in the iPOC variation within migratory marine mammals and seabirds (Fig. 2d). For example, high levels of iPOC (>50%) were observed in the summer and winter sampled beluga whales (*Delphinapterus leucas)* from Cumberland Sound (*n* = 142 of 155), but we observed low iPOC levels (<25%) in nearly all samples collected in July from the eastern Beaufort Sea (*n* = 21 of 22). This may be a result of the Cumberland Sound beluga population that do not migrate and remain in icy Arctic environments year-round^36^, while the eastern Beaufort Sea population have long migrations and spend time foraging further south in marginal ice waters in the subarctic Bering and Chukchi Seas^68^. Additionally, similar variations in iPOC values were observed in Pacific walrus populations, which have largely sex-segregated migrations. The males remain in the Bering Sea year-round and reflected in low iPOC values, while the females and calves migrate with the ice edge north into the Chukchi Sea in the spring and summer, where we observed higher iPOC values^35^. Similarly in seabird populations, thick-billed murres (*Uria lomvia*) from Prince Leopold Island in Nunavut indicated greater reliance on ice algal carbon sources and sensitivity to changes in sea ice on an annual basis compared to northern fulmars (*Fulmaris glacialis*), which have a greater foraging area less constrained by sea ice conditions^37^.

### Stored sea-ice carbon fueling winter ecosystems

In contrast to the established understanding that springtime is known for its significant productivity in the Arctic^1^, until relatively recently, it was assumed the dark Arctic winters were a period of biological dormancy^69^. We now find this idea is being challenged as wintertime studies are increasingly revealing significant biomass and biological activity^69–72^, despite extremely low levels of in situ primary production^61,73^. While sources of organic carbon, other than locally produced marine primary production, are available in the Arctic, including advected temperate phytoplankton^61,74^, terrestrial river discharges^75^, benthic algae^76^, or even recycled carbon from seabird guano^77^ or marine mammal defecation^78^, these are relatively localized. In contrast, sea-ice primary production is widespread^79^.

**Fig. 4:**
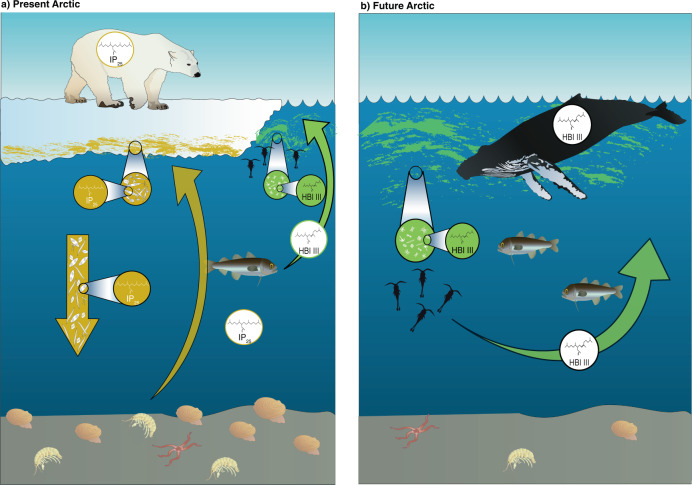
Schematic illustration of Arctic shelf sea ice-benthic coupling under current and future conditions. **a** The transfer of the sea-ice carbon, signified by the diatom biomarker IP_25_^11^ through the food web via sinking sea-ice algae, results in ‘sea ice-benthic coupling’ and the subsequent ‘bottom-up’ transfer of carbon from diverse seafloor fauna into pelagic consumers, in addition to the pelagic phytoplankton bloom signified by the diatom biomarker HBI III^9^. **b** A future pelagic-dominated Arctic shelf scenario with extended open-water periods, ice-free summers and delayed blooms. The pulsative delivery of sea-ice carbon to the seafloor is removed and replaced exclusively by the transfer of pelagic carbon (as indicated by HBI III). Algal community composition has shifted, including introduction of new species and reduced biomass and abundance, as a result of these changes in the food web. Symbols courtesy of the Integration and Application Network (ian.umces.edu/media-library) licensed under CC BY-SA 4.0 (polar bear, humpback whale, mixed phytoplankton, pennate diatom, *Gammarus* amphipod, clam – Tracey Saxby; *Arctogadus glacialis* – Kim Kraeer, Lucy Van Essen-Fischer; diatoms – Diana Kliene, Marine Botany UQ).

We suggest the primary mechanism driving the availability of sea-ice organic carbon year-round is benthic heterotrophs feeding on sedimentary stores of sea-ice organic carbon deposited during the spring bloom (Fig. 4). Additionally, fluxes of sympagic HBIs were observed year-round on the highly productive northeast Chukchi shelf^[ 80^, and may occur at other locations on Arctic shelves. In support of this, we show that iPOC is present in winter (October – March) sampled animals (Fig. 2) where it represents a comparable proportion of carbon (relative to pelagic primary production) to that measured in springtime sampled animals. It is unlikely that this is caused by the retention of HBIs in animal tissues, similar to contaminants^81^, as this has not yet been observed in marine animals^15,36,64,65^, suggesting the sea-ice organic carbon we measure in animal tissues here was acquired recently (<1 month prior to sampling). Feeding strategy likely also influences the amount of iPOC observed in benthic invertebrates (Fig. 2a), where surface deposit feeders have prolonged access to freshly deposited ice algae and suspension feeders would have HBI values reflecting the most recent flux of organic matter and thus more variable^31^. The predators and scavengers indicate high utilization of iPOC via benthic prey, while subsurface deposit feeders had the lowest mean iPOC but with a wide range of values.

**Fig. 5:**
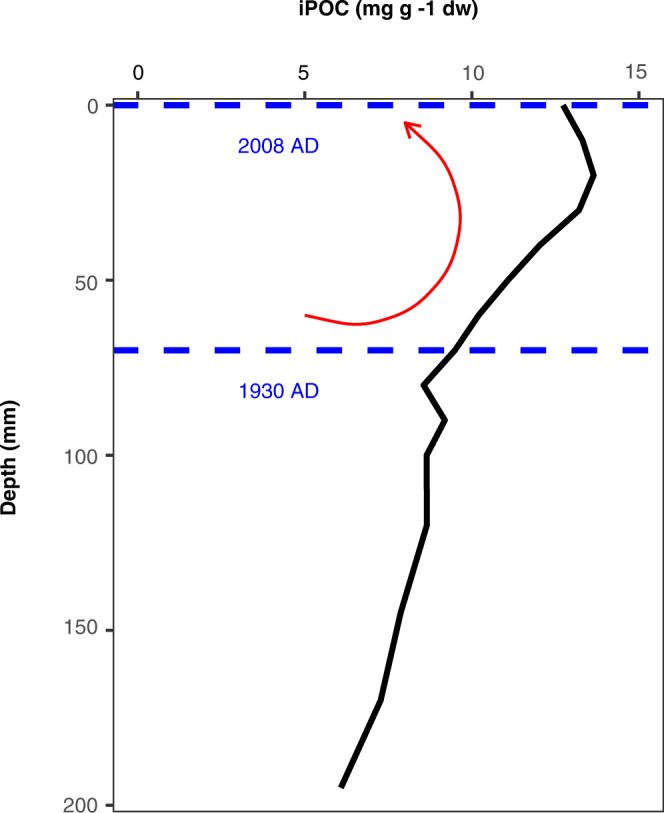
Maximum theoretical estimate of sea ice-derived carbon in marine sediments in the shallow Southeast Beaufort Sea shelf region. Black line shows estimated sea-ice organic carbon (iPOC) concentration. Horizontal blue lines show Pb^210^ dates corresponding to sampling (upper) and 70 mm (lower). Red arrow indicates region of bioturbation potential and accessible iPOC.

To illustrate the availability of sea ice-derived carbon in sediments, we estimated the iPOC content of Arctic surface sediments using previously measured source specific biomarkers (recalculated here as iPOC) and total organic carbon from a box core collected in the Amundsen Gulf (Station 405b; 70°40 N, 123°00 W; 500 m depth) in 2008 as part of the International Polar Year – Circumpolar Flaw Lead study^82^. Accordingly, we show that for the shallow Southeast Beaufort Sea shelf region, a theoretical maximum of 9–14 mg of iPOC was deposited per gram (dry weight) of sediment within the upper 70 mm (Fig. 5). With sedimentation rates of approximately 1 mm y^−1^ ^82^, infauna capable of burrowing up to 70 mm^83^ may have access to sea- ice carbon deposited throughout the last century^84^. We accept that the details of this observation are likely to vary regionally and do not account for the rapid and high degree of bioturbation on Arctic shelves that contribute to the burial of unused lipid reserves^84^. Nevertheless, these estimates identify that sea-ice organic carbon will still be present within the marine ecosystem for a number of years following significant sea-ice decline, potentially buffering the true impact of the loss of sea ice up to a certain point on the long-term sustainability of the future Arctic ecosystem.

### Consequences for a changing Arctic

The Arctic is rapidly changing, with recent estimates suggesting a warming upwards of four times faster than the rest of the planet^85^. Changes we are already witnessing include the shrinking and thinning of multiyear ice leading to greater areas of open water in summer^86^. Recent observations suggest that over the last several decades, this thinning of sea ice has resulted in greater potential habitat for ice algae in the near term^87^. Even if temporarily expanding, the redistribution of this habitat has major implications for underlying benthic ecosystems relying on this resource. A reversal of this trend in increasing first-year sea ice is expected in the coming decades with the gradual disappearance of seasonal ice in summer. Changes in the phenology and duration of Arctic sea-ice melt are also evident, with seasonal ice in some sub-Arctic regions melting up to seven days earlier each year^88^. The reduction of sea-ice habitat, currently occurring at the lower sub-Arctic regions (e.g., Bering Sea), is suggested to result in a decoupling of sea-ice and benthic habitats. It is, therefore unclear if a future Arctic, with no summer sea ice and potentially reduced, or even absent, sea ice-benthic coupling in at least some regions, will provide sufficient primary production to sustain the sympagic ecosystem throughout the extended dark winter period. In a summer without sea ice, phytoplankton will be highly productive (and be rapidly removed by specialist pelagic consumers, particularly seasonal migrants), resulting in partial remineralization in the water column and the remaining refractory organic matter settling to the seafloor. We clearly show here that a large component of the Arctic marine ecosystem relies on benthic carbon storage, adapted to ice algae production that allows for recycling through the winter dark period. Consequently, the major ecosystem changes will be twofold – first we will see a mismatch in life histories adapted to this carbon source that have evolved with seasonal sea ice; and second, there will be a redistribution of species and their habitats to respond to this shifting carbon source eventually leading to reduced Arctic and global biodiversity.

It is difficult to accurately predict the type of Arctic ecosystem that will evolve without sea ice – one that depends on a large summer phytoplankton production and no stored energy during the winter. While the above mentioned changes in sea ice and the resulting extended open water period may provide a setting for a longer phytoplankton growth season, this must be accompanied by increases in the springtime nutrient load that can be sustained through summer^39^. Indeed, the ability of an extended phytoplankton season to suitably subsidize a loss of sea-ice organic carbon would likely depend upon a sufficient excess primary production that continues exceeding the grazing potential of pelagic consumers. Some suggest a more than three-fold increase in pelagic primary production is possible^89^, and that an increased proportion of thinner seasonal sea ice and/or less snow cover can transmit sufficient light for under-ice phytoplankton blooms to begin even before ice has melted^90,91^. There may also be an associated decline in ice algae and phytoplankton biodiversity and size^92^, outside the preferred size range of some primary consumers. However, these outcomes depend, in part, on sufficient over-winter nutrient availability and the ability of temperate-associated species to move in and compete for resources. Perhaps then, in the event that the availability of sea ice-algae is greatly reduced and phytoplankton cannot match the current capacity for year-round energy supply, the benthic retention of sea-ice carbon will not be sufficient to support organisms throughout the winter. The resulting poor understanding in organic carbon contributions has, in part, led to the Arctic Nations taking a proactive approach in declaring a moratorium on commercial fishing in Arctic high seas for a period of sixteen years, until 2037^93^. Intended to protect Arctic marine species from potential overexploitation, this has also highlighted the urgent need for research into the future sustainability of the Arctic ecosystem in the face of continued climate warming and reducing sea ice^94^. This further emphasizes the critical role of and urgent need for increased biological monitoring programs and proactive approaches for sustainable commercial activities to allow a transition that minimizes species loss.

## Methods

### Sample collection

A majority of samples and associated HBI data analyzed for this study were reported in previously published studies and recalculated as iPOC, including several datasets that were not included in the original publications (provided by the respective authors; Supplementary Table 1). In general, marine mammal samples consisted of liver tissues collected from subsistence harvesting activities and were donated (see original cited references for species-specific details, Supplementary Table 1). Fish, zooplankton, and benthic invertebrate samples were live collected on various research cruises and stored frozen at −80 °C. Seabird samples were measured from eggs collected from northern fulmar and thick-billed murre nests that were kept cool in the field and through shipment until processing. Egg contents were then homogenized and stored frozen at −40 °C^37^.

### Extraction and analysis of highly branched isoprenoid (HBI) lipids

HBI extraction and analysis followed established protocols^95^. Liver tissue of fishes and marine mammals, homogenized seabird egg contents, and whole samples of invertebrates, were freeze-dried and saponified (~ 5 mL H_2_O:MeOH, 1:9; 20% KOH; 60 mins; 70 ^o^C), mixed with hexane (3 ×4 mL), then centrifuged (2 min; 1048  ×  *g*; repeated three times). The hexane layer is then transferred to a clean vial and dried under a gentle N_2_ stream. Dried lipid extracts were resuspended and fractionated (5 mL hexane) using open-column silica gel chromatography (SiO_2_; 0.5 g). The non-polar lipids containing the HBIs were eluted while the polar compounds were retained on the column. The eluted compounds were dried under N_2_. 50 μL of hexane was added twice to the dried extract and transferred to amber chromatography vials for analysis.

HBIs were analyzed by gas chromatography–mass spectrometry on an Agilent HP-5 ms column (30 m × 0.25 mm × 0.25 μm). The oven temperature was programmed to ramp up from 40 °C to 300 °C at 10 °C/min with a 10 min isothermal period at 300 °C. HBIs were identified and quantified by measuring the retention indices and mass spectral intensities for each HBI in selective ion monitoring (SIM) mode^36^. The HBIs identified and quantified included IP_25_ (m/z 350.3), HBI II (m/z 348.3) and HBI III (m/z 346.3).

### Sympagic carbon estimates

Estimates of iPOC were based on the H-Print which was calculated using the analytical intensities of three HBIs (IP_25_: *m/z* 350.3, II; *m/z* 348.3 and III; *m/z* 346.3), according to Eq. 1, since this combination enabled a linear calibration to be constructed previously^8^.

1 $${{{{{\rm{H}}}}}}{\mbox{-}}{{{{{\rm{Print}}}}}} \, (\%)=\frac{\left({{{{{\rm{HBI\; III}}}}}}\right)}{\left({{{{{{\rm{IP}}}}}}}_{25}+{{{{{\rm{HBI\; II}}}}}}+{{{{{\rm{HBI\; III}}}}}}\right)}\times 100.$$

Sea-ice carbon, as a proportion of marine-origin carbon within samples, was estimated using Eq. 2 from previous H-Print calibration (R^2^ = 0.97, *P* = < 0.01, df =  23^8^). This equation was based on a controlled feeding experiment using ice algae with known quantities of HBI II. HBI concentrations were measured throughout the experiment. The test organisms were ensured to have depurated any natural occurrence of HBIs before being fed the ice algae and measured repeatedly until all HBIs had been eliminated. Sea-ice carbon (iPOC) estimates are expressed here as mean values.

2 $${{{{{\rm{iPOC}}}}}} \, \left(\%\right)=101.08{{{{{\rm{\hbox{-}}}}}}}1.02 \, {{{{{\rm{x}}}}}} \, {{{{{\rm{H}}}}}}{\mbox{-}}{{{{{\rm{Print}}}}}}.$$

### Statistical analysis

Organisms were classified by their primary habitat and feeding strategies (benthic invertebrates only) using available resources, including various online species databases^96–99^ and publications^19,100–102^. All analyses were conducted using R version 4.2.2 (www.r-project.org) and plots were produced using the package ‘ggplot2’. A significance level of α = 0.05 was used for all tests. Our model was designed with the following assumptions based on prior knowledge, 1) there are multiple confounders that can influence iPOC including time of sampling, time of ice retreat relative to sample location, feeding strategies, nutrient availability, ice algae species composition and production, and varying HBI retention time of individuals, 2) iPOC can vary widely between and within species, 3) all samples were collected north of the median maximum sea-ice extent and would therefore have the potential to consume ice algae or sea-ice derived carbon sources. Multiple linear regressions were used to explore correlations between sea ice algae carbon estimates and several predictor variables (month, year, species, taxa, habitat, latitude) and applicable interactions (month*habitat). AIC scores of each model were compared using package ‘AICcmodavg’. The model with the lowest AIC score (iPOC ~ month+latitude+ habitat+month*habitat) was further assessed using the ‘sensemakr’ sensitivity analysis package^103^ with habitat as the treatment and month as the confounder, as sea-ice retreat generally follows a pattern one could anticipate whether there would be open water (summer) or sea ice (winter). Samples without month recorded were excluded (*n* = 65). The partial R^2^ of habitat with the outcome was 0.02 and the robustness value was 0.05 at α = 0.05. We further explored the sensitivity by shifting each observation three months earlier. The result was similar to our original model, suggesting that there is minimal effect of any one of these environmental parameters alone in explaining the variability observed in our iPOC values, but is rather likely a combination of factors.

## Supplementary information

Supplementary Information

## Data Availability

The estimated iPOC data summarised by species and contributing author are available in the Supplementary Information with associated references. The individual sea ice organic carbon estimates (iPOC%) and model fit data generated in this study have been deposited in the Arctic Data Center repository [10.18739/A2D50FZ9W].
