# Supplementary material for: Year-round utilization of sea ice-associated carbon in Arctic ecosystems
Source: Nat Commun. 2023 Apr 7;14:1964. doi: 10.1038/s41467-023-37612-8 (PMC10081986; doi:10.1038/s41467-023-37612-8)
Supplement: Supplementary file 1 — Supplementary Information [file 41467_2023_37612_MOESM1_ESM.pdf]

Supplementary Information: Year-round utilization of sea ice-associated carbon in Arctic ecosystems

Chelsea W. Koch[1,2], Thomas A. Brown[3], Rémi Amiraux[4], Carla Ruiz-Gonzalez[3], Maryam Maccorquodale[3], Gustavo A. Yunda-Guarin[5], Doreen Kohlbach[6], Lisa L. Loseto[7], Bruno Rosenberg[7], Nigel E. Hussey[8], Steve H. Ferguson[7], David J. Yurkowski[7]

Affiliations:

[1] Natural History Museum, London, SW7 5BD, England

[2] University of Maryland Center for Environmental Science, Solomons, Maryland, United States

[3] Scottish Association for Marine Science, Oban, PA37 1QA, Scotland

[4] Centre for Earth Observation Science, University of Manitoba, Winnipeg, Canada

[5] Québec-Océan and Takuvik, Biology Department, Laval University, Québec, Quebec, Canada

[6] Norwegian Polar Institute, Fram Centre, Tromsø 9296, Norway

[7] Fisheries and Oceans Canada, Freshwater Institute, Winnipeg, Canada

[8] Integrative Biology, University of Windsor, Windsor, Ontario, N9B 3P4, Canada

Supplementary Table 1 – Mean estimated sea ice particulate organic carbon (iPOC %) values and statistics [maximum values (Max), minimum values (Min), standard deviation (SD), standard error (SE) and sample size (n)] grouped by species and individual contributor (author initials). Additional sample data were contributed courtesy of Philipp Assmy (Norweigan Polar Institute), L. Winberg (University of Gothenburg), Dieter Piepenburg (Alfred Wegener Institute), Fanny Cusset (La Rochelle University), and Markus G. Dyck (Government of Nunavut). The source of original highly branched isoprenoid (HBI) values used to estimate iPOC are provided under the “source” column (see reference list at bottom of table). Data that have not been previously published are noted as “new”. Individual rows are listed by species (or lowest possible taxonomic group possible) and if available, a common name is provided.

**Species**	**Common Name**	**Mean estimated iPOC%**	**Max**	**Min**	**SD**	**SE**	**n**	**Contributor**	**Source**
Actinauge cristata		69.05	89.52	53.78	17.26	8.63	4	G.Y.G.	1
Aglaophamus malmgreni		89.34	89.34	89.34			1	G.Y.G.	1
Ampelisca sp.		8.47	37.06	-1.08	14.88	4.49	11	C.W.K.	2
Anonyx nugax		77.88	77.88	77.88			1	T.A.B.	3
Anonyx nugax		84.07	84.07	84.07			1	G.Y.G.	1
Apherusa glacialis		92.22	101.08	37.68	18.59	4.80	15	T.A.B. (Philipp Assmy)	4
Arctogadus glacialis	Ice or Arctic cod	12.35	19.38	7.67	3.49	0.87	16	S.H.F.	New
Aspidophoroides olrikii	Alligator Fish	36.22	53.79	17.11	15.80	6.45	6	N.E.H.	New
Astarte borealis		12.93	38.90	0.75	17.95	8.98	4	C.W.K.	2
Astarte crenata		37.61	38.99	36.22	1.96	1.39	2	G.Y.G.	1
Astarte sp.		92.33	93.29	91.36	1.36	0.96	2	T.A.B.	3
Astarte sp.		53.94	53.94	53.94			1	G.Y.G.	1
Astarte sp.		84.71	86.01	83.40	1.84	1.30	2	T.A.B. (Winberg)	New
Bathybiaster vexillifer		89.70	91.12	88.28	2.01	1.42	2	G.Y.G.	1
Bathylagus euryops	goiter blacksmelts	54.91	76.06	36.08	12.67	3.17	16	N.E.H.	New
Bathypolypus bairdii		57.39	57.39	57.39			1	G.Y.G.	1
Beroe cucumis		-1.08	-1.08	-1.08			1	D.K.	5
Bolocera tuediae		58.07	58.73	57.40	0.94	0.67	2	G.Y.G.	1
Boreogadus saida	Polar cod	7.48	15.07	3.03	3.50	0.94	14	D.J.Y. & R.A.	6
Boreogadus saida	Polar cod	73.85	73.85	73.85			1	T.A.B.	3
Boreogadus saida	Polar cod	44.51	101.08	4.46	33.94	5.66	36	S.H.F.	New
Boreogadus saida	Polar cod	19.35	64.89	-1.08	11.39	0.98	135	N.E.H.	New
Boreomysis nobilis		96.70	98.04	95.37	0.85	0.30	8	G.Y.G.	1
Bougainvillia superciliaris		-1.08	-1.08	-1.08			1	D.K.	5
Buccinidae		88.89	97.44	80.54	5.55	1.60	12	T.A.B. (Winberg)	New
Calanus finmarchicus		-1.08	-1.08	-1.08	0.00	0.00	6	D.K.	5, 7
Calanus glacialis		-1.08	-1.08	-1.08	0.00	0.00	5	D.K.	5, 7

Calanus hyperboreus		-1.08	-1.08	-1.08	0.00	0.00	5	D.K.	5, 7
Catablema visicarium		-1.08	-1.08	-1.08			1	D.K.	5
Ceramaster granularis		56.66	65.32	43.28	11.75	6.79	3	G.Y.G.	1
Chaetognatha		34.23	61.19	7.41	19.15	4.51	18	D.J.Y. & R.A.	6
Chionoecetes opilio		9.53	11.99	7.07	3.48	2.46	2	C.W.K.	2
Chlamys islandica		95.39	95.95	94.84	0.79	0.56	2	T.A.B. (Winberg)	New
Clinocardium ciliatum		57.53	76.53	46.32	11.49	5.14	5	T.A.B. (Winberg)	New
Clione limacina		101.08	101.08	101.08	0.00	0.00	2	D.K.	5
Clione limacina		4.86	8.69	2.14	2.16	0.77	8	D.J.Y. & R.A.	6
Coregonus autumnalis	Arctic Cisco	97.60	100.20	93.83	1.90	0.43	20	L.L.	New
Coregonus nasus	Broad Whitefish	101.05	101.07	100.91	0.04	0.01	19	L.L.	New
Cottoidea	Sculpin	89.44	98.30	76.44	5.93	1.33	20	S.H.F.	New
Cottoidea	Sculpin	71.55	93.00	45.55	13.09	3.50	14	N.E.H.	New
Cryptonatica clausa		93.77	97.57	87.27	3.45	1.15	9	T.A.B. (Winberg)	New
Ctenodiscus crispatus		61.56	62.02	61.09	0.66	0.47	2	G.Y.G.	1
Ctenophore		36.64	42.93	30.34	8.90	6.30	2	D.J.Y. & R.A.	6
Cuspidaria glacialis		66.53	68.24	64.83	2.41	1.71	2	G.Y.G.	1
Delphinapterus leucas	Beluga whale	76.56	100.62	14.66	17.87	1.32	184	S.H.F. & D.J.Y.	8
Delphinapterus leucas	Beluga whale	18.27	50.30	9.06	7.29	1.29	32	L.L.	New
Diastylis rathkei		67.75	82.40	43.06	21.50	12.41	3	G.Y.G.	1
Drifa glomerata		66.13	66.13	66.13			1	G.Y.G.	1
Eleginus gracilis	Saffron Cod	68.60	96.91	42.65	16.89	3.78	20	L.L.	New
Ennucula tenuis		28.40	76.66	0.16	25.91	6.11	18	C.W.K.	2
Erignathus barbatus	Bearded Seal	96.88	96.88	96.88			1	S.H.F.	New
Eumicrotremus derjugini	leatherfin lumpsucker	11.12	11.12	11.12			1	N.E.H.	New
Eusirus holmi		101.08	101.08	101.08	0.00	0.00	14	T.A.B. (Philipp Assmy)	4
Fulmaris glacialis	Northern fulmar	56.09	101.08	-1.08	32.83	4.27	59	F.C.	9
Gadus morhua	Atlantic cod	58.53	58.53	58.53			1	T.A.B.	3
Gamarus wilkitzkii		98.92	101.08	93.79	2.19	0.38	34	T.A.B. (Philipp Assmy)	4
Geodia macandrewii		56.32	58.30	54.34	2.80	1.98	2	G.Y.G.	1
Golfingia margaritacea		41.45	77.07	15.47	22.52	9.19	6	C.W.K.	2
Gorgonocephalus lamarckii		63.26	84.83	48.81	17.00	8.50	4	G.Y.G.	1
Gorgonocephalus sp.		20.09	20.09	20.09			1	C.W.K.	2
Hanleya hanleyi		54.74	56.44	53.05	2.40	1.70	2	G.Y.G.	1
Henricia sp.		67.97	85.91	53.49	13.62	6.09	5	G.Y.G.	1
Henricia sp.		50.21	50.21	50.21			1	C.W.K.	2

Hiatella arctica		80.88	83.76	77.99	4.08	2.89	2	T.A.B. (Winberg)	New
Hippasteria phrygiana		16.12	16.12	16.12			1	G.Y.G.	1
Hippoglossoides platessoides	American plaice	31.68	31.68	31.68			1	G.Y.G.	1
Hippoglossoides platessoides	American plaice	19.63	19.63	19.63			1	N.E.H.	New
Hyas coarctatus		78.58	78.58	78.58			1	T.A.B.	10
Hydrozoa		34.37	54.82	20.71	13.35	5.04	7	D.J.Y. & R.A.	6
Hymenaster pellucidus		82.67	82.67	82.67			1	G.Y.G.	1
Hymenodora glacialis		93.26	98.91	90.29	2.71	1.03	7	G.Y.G.	1
Icasterias panopla		57.01	57.44	56.58	0.61	0.43	2	G.Y.G.	1
Icelus bicornis	Twohorn sculpin	69.23	69.23	69.23			1	T.A.B.	3
Jasmineira schaudinni		89.85	95.40	79.81	5.70	2.33	6	G.Y.G.	1
Lebbeus polaris		60.35	93.93	24.30	26.28	9.29	8	G.Y.G.	1
Leptagonus decagonus	Atlantic poacher	48.50	48.50	48.50			1	T.A.B.	3
Leptychaster arcticus		75.70	75.70	75.70			1	G.Y.G.	1
Limacina helicina		96.20	101.08	91.32	6.90	4.88	2	D.K.	5
Liparidae	Snail Fish	7.26	71.57	-1.08	14.76	2.25	43	N.E.H.	New
Liparis fabricii	Gelatinous snailfish	37.00	37.00	37.00			1	T.A.B.	10
Liparis gibbus	Variegated snailfish	46.91	46.91	46.91			1	T.A.B.	3
Liponema multicorne		89.68	89.96	89.40	0.40	0.28	2	G.Y.G.	1
Lithodes maja		68.65	68.65	68.65			1	G.Y.G.	1
Lumbrineris sp.		22.08	22.08	22.08			1	C.W.K.	2
Lumpenus lampretaeformis	Snakeblenny	26.27	26.27	26.27			1	G.Y.G.	1
Lycodes esmarkii	Greater eelpout	76.22	76.22	76.22			1	T.A.B.	New
Macoma sp.		15.16	59.64	-1.08	18.06	3.10	34	C.W.K.	2
Maldanidae		27.89	78.35	-1.08	25.86	6.09	18	C.W.K.	2
Mallotus villosus	Capelin	15.47	15.47	15.47			1	T.A.B.	8
Mallotus villosus	Capelin	34.82	63.20	14.17	15.15	5.36	8	N.E.H.	8
Meganyctiphanes norvegica		6.49	29.18	-1.08	15.13	7.56	4	D.K.	7
Melanogrammus aeglefinus	Haddock	73.66	73.66	73.66			1	T.A.B.	New
Mertensia ovum		-1.08	-1.08	-1.08			1	D.K.	5
Metridia longa		-1.08	-1.08	-1.08	0.00	0.00	2	D.K.	5
Molpadia sp.		72.64	72.64	72.64			1	G.Y.G.	1
Monodon monoceros	Narwhal	79.65	83.71	74.15	4.10	1.84	5	S.H.F.	New
Musculus sp.		11.19	22.51	-0.14	16.02	11.33	2	C.W.K.	2
Mya sp.		3.18	3.18	3.18			1	C.W.K.	2
Mya truncata		4.28	4.29	4.28	0.01	0.00	2	C.W.K.	2
Mya truncata		92.41	92.41	92.41			1	T.A.B. (Winberg)	New

Myriotrochus sp.		22.26	22.26	22.26			1	C.W.K.	2
Mysis oculata		18.05	23.40	14.33	3.93	1.97	4	D.J.Y. & R.A.	6
Nephtys sp.		8.24	23.44	-1.08	7.41	2.62	8	C.W.K.	2
Nuculana pernicula		20.45	39.16	1.74	26.46	18.71	2	C.W.K.	2
Nutricola sp.		3.65	8.38	-1.08	6.69	4.73	2	C.W.K.	2
Odobenus rosmarus divergens	Pacific walrus	37.22	94.90	-0.90	22.45	1.89	141	C.W.K.	11
Odobenus rosmarus rosmarus	Atlantic walrus	68.07	100.30	1.77	27.50	3.61	58	D.J.Y.	12
Oikopleura vanhoeffeni		101.08	101.08	101.08			1	D.K.	5
Onisimus glacialis		101.08	101.08	101.08	0.00	0.00	10	T.A.B. (Philipp Assmy)	4
Onisimus nanseni		100.74	101.08	98.97	0.66	0.18	13	T.A.B. (Philipp Assmy)	4
Ophiacantha bidentata		86.71	86.76	86.66	0.07	0.05	2	G.Y.G.	1
Ophiacantha spectabilis		77.48	79.65	75.77	1.69	0.84	4	G.Y.G.	1
Ophiocten sericeum		38.54	54.51	8.34	26.17	15.11	3	C.W.K.	2
Ophiopholis aculeata		81.54	90.73	62.54	13.06	6.53	4	G.Y.G.	1
Ophiopleura borealis		94.70	99.33	83.56	7.46	3.73	4	G.Y.G.	1
Ophioscolex glacialis		84.96	84.96	84.96			1	G.Y.G.	1
Ophiura sarsii		89.02	91.08	85.05	3.44	1.99	3	G.Y.G.	1
Osmerus mordax	Rainbow smelt	53.40	83.83	37.82	14.63	4.88	9	L.L.	New
Pagophilus groenlandicus	Harp Seal	68.56	91.90	26.28	24.42	8.63	8	S.H.F.	New
Pagurus pubescens		24.51	24.51	24.51			1	G.Y.G.	1
Pandalus borealis		74.76	86.78	62.74	17.00	12.02	2	G.Y.G.	1
Pandalus borealis		68.88	73.92	58.85	8.69	5.01	3	T.A.B.	10
Pandora sp.		2.88	2.88	2.88			1	C.W.K.	2
Paraeuchaeta glacialis		-1.08	-1.08	-1.08	0.00	0.00	3	D.K.	7
Paraeuchaeta norvegica		-1.08	-1.08	-1.08			1	D.K.	5
Pectinaria hyperborea		30.99	66.67	5.08	25.42	7.34	12	C.W.K.	2
Phoca vitulina	Harbour seal	67.69	80.85	45.24	14.36	5.86	6	S.H.F.	New
Phyllodoce groenlandica		71.81	71.81	71.81			1	T.A.B.	New
Platichthys stellatus	Starry Flounder	99.81	100.95	95.60	1.26	0.27	22	L.L.	New
Polymastia hemisphaerica		65.19	65.19	65.19			1	G.Y.G.	1
Polymastia sp.		74.92	74.92	74.92			1	G.Y.G.	1
Polynoidae		78.09	78.09	78.09			1	T.A.B.	3
Polyphysia crassa		83.52	84.47	81.32	1.28	0.57	5	G.Y.G.	1
Pontaster tenuispinus		94.42	96.69	90.38	2.40	1.07	5	G.Y.G.	1
Porifera		95.71	95.71	95.71			1	G.Y.G.	1
Pteropod		7.74	9.03	6.08	0.94	0.28	11	D.J.Y. & R.A.	6
Ptilella grandis		14.05	14.05	14.05			1	S.H.F.	New
Ptilella grandis		61.96	87.49	45.83	22.37	12.91	3	G.Y.G.	1

Pusa hispida	Ringed Seal	92.54	101.08	10.07	13.07	0.56	546	S.H.F.	13, 14
Reinhardtius hippoglossoides	Greenland halibut	74.87	89.55	39.46	8.14	1.11	54	N.E.H.	8
Rossia megaptera		81.52	84.38	78.66	4.04	2.86	2	G.Y.G.	1
Rossia sp.		66.95	68.15	65.75	1.70	1.20	2	G.Y.G.	1
Sabinea sarsii		80.90	80.90	80.90			1	G.Y.G.	1
Sagitta elegans		-1.08	-1.08	-1.08	0.00	0.00	2	D.K.	5
Sclerocrangon ferox		69.33	69.33	69.33			1	G.Y.G.	1
Sebastes mentella	Red fish	14.47	14.47	14.47			1	T.A.B.	New
Sebastes mentella	Red fish	53.01	53.01	53.01			1	N.E.H.	New
Serripes groenlandicus		86.77	98.71	25.80	16.76	2.16	60	T.A.B. (Winberg)	New
Serripes lamperosii		0.19	2.74	-1.08	1.80	0.80	5	C.W.K.	2
Spirontocaris liljeborgii		53.38	80.16	26.61	37.87	26.78	2	G.Y.G.	1
Stenosemus albus		60.19	87.60	32.79	38.76	27.40	2	G.Y.G.	1
Stephanasterias albula		60.59	66.17	53.31	5.10	1.93	7	G.Y.G.	1
Strongylocentrotus droebachiensis		84.19	84.89	83.49	0.99	0.70	2	G.Y.G.	1
Strongylocentrotus sp.		95.87	95.87	95.87			1	T.A.B.	15
Strongylocentrotus sp.		85.96	91.81	80.96	3.19	0.92	12	T.A.B. (Findlay)	15
Strongylocentrotus sp.		82.88	100.83	5.88	25.73	4.55	32	T.A.B. (Dieter Piepenburg)	New
Styela rustica		13.75	13.75	13.75			1	C.W.K.	2
Themisto abyssorum		90.75	90.75	90.75			1	G.Y.G.	1
Themisto abyssorum		62.77	101.08	39.16	22.33	9.12	6	D.K.	5, 7
Themisto libellula		33.62	79.88	-1.08	28.20	9.97	8	D.K.	5, 7
Themisto libellula		9.08	18.56	-0.11	8.20	3.35	6	D.J.Y. & R.A.	6
Thenea muricata		83.33	83.33	83.33			1	G.Y.G.	1
Thysanoessa inermis		18.02	31.12	-1.08	13.55	4.79	8	D.K.	5
Thysanoessa inermis		9.51	9.51	9.51			1	D.J.Y. & R.A.	6
Thysanoessa raschii	Arctic krill	30.46	50.97	13.17	17.28	8.64	4	D.J.Y. & R.A.	6
Thysanoessa sp.		-1.08	-1.08	-1.08	0.00	0.00	2	D.K.	5
Tremaster mirabilis		60.16	60.16	60.16			1	G.Y.G.	1
Triglops nybelini	Crocodile fish	37.83	64.39	-1.08	24.01	7.59	10	N.E.H.	New
Triglops pingeli	Ribbed sculpin	13.94	54.27	-1.08	17.96	5.68	10	N.E.H.	New
Urasterias lincki		38.58	38.58	38.58			1	G.Y.G.	1
Uria lomvia	Thick-billed murre	42.09	93.87	2.72	29.39	3.79	60	F.C.	9
Ursus maritimus	Polar bear	86.91	100.53	34.23	14.82	1.54	93	T.A.B. (Markus G. Dyck)	16
Yoldia hyperborea		28.85	68.67	1.74	27.57	8.31	11	C.W.K.	2
Yoldiella lenticula		95.19	95.19	95.19			1	G.Y.G.	1
Zoarcidae	Eel Pout	46.24	46.24	46.24			1	N.E.H.	New

References

1. Yunda-Guarin G, *et al.* Reliance of deep-sea benthic macrofauna on ice-derived organic matter highlighted by multiple trophic markers during spring in Baffin Bay, Canadian Arctic. *Elementa: Science of the Anthropocene* 8, (2020). DOI: https://doi.org/10.1525/elementa.2020.047

2. Koch CW, Cooper LW, Grebmeier JM, Frey K, Brown TA. Ice algae resource utilization by benthic macro-and megafaunal communities on the Pacific Arctic shelf determined through lipid biomarker analysis. *Marine Ecology Progress Series* 651, 23-43 (2020). DOI: https://doi.org/10.3354/meps13476

3. Brown T, Hegseth E, Belt S. A biomarker-based investigation of the mid-winter ecosystem in Rijpfjorden, Svalbard. *Polar Biology* 38, 37-50 (2015). DOI: https://doi.org/10.1007/s00300-013-1352-2

4. Brown TA, Assmy P, Hop H, Wold A, Belt ST. Transfer of ice algae carbon to ice-associated amphipods in the high-Arctic pack ice environment. *Journal of Plankton Research* 39, 664-674 (2017). DOI: https://doi.org/10.1093/plankt/fbx030

5. Kohlbach D, *et al.* Multiple trophic markers trace dietary carbon sources in Barents Sea zooplankton during late summer. *Frontiers in Marine Science* 7, 610248 (2021). DOI: https://doi.org/10.3389/fmars.2020.610248

6. Amiraux R, *et al.* Tracing carbon flow and trophic structure of a coastal Arctic marine food web using highly branched isoprenoids and carbon, nitrogen and sulfur stable isotopes. *Ecological Indicators* 147, 109938 (2023). DOI: https://doi.org/10.1016/j.ecolind.2023.109938

7. Kohlbach D, *et al.* Winter Carnivory and diapause counteract the reliance on ice algae by Barents Sea zooplankton. *Frontiers in Marine Science*, 266 (2021). DOI: https://doi.org/10.3389/fmars.2021.640050

8. Brown TA, *et al.* Coupled changes between the H-Print biomarker and $\delta^{15}N$ indicates a variable sea ice carbon contribution to the diet of Cumberland Sound beluga whales. *Limnology and Oceanography* 62, 1606-1619 (2017). DOI: https://doi.org/10.1002/lno.10520

9. Cusset F, Fort J, Mallory M, Braune B, Massicotte P, Másse G. Arctic seabirds and shrinking sea ice: egg analyses reveal the importance of ice-derived resources. *Scientific reports* 9, 1-15 (2019). DOI: https://doi.org/10.1038/s41598-019-51788-4

10. Brown TA, Belt ST. Closely linked sea ice-pelagic coupling in the Amundsen Gulf revealed by the sea ice diatom biomarker IP_{25}. *Journal of Plankton Research* 34, 647-654 (2012). DOI: https://doi.org/10.1093/plankt/fbs045

11. Koch CW, *et al.* Female Pacific walruses (*Odobenus rosmarus divergens*) show greater partitioning of sea ice organic carbon than males: Evidence from ice algae trophic markers. *PLOS ONE* 16, e0255686 (2021). DOI: https://doi.org/10.1371/journal.pone.0255686

12. Yurkowski DJ, Brown TA, Blanchfield PJ, Ferguson SH. Atlantic walrus signal latitudinal differences in the long-term decline of sea ice-derived carbon to benthic fauna in the Canadian Arctic. *Proceedings of the Royal Society B* 287, 20202126 (2020). DOI: https://doi.org/10.1098/rspb.2020.2126

13. Brown T, Alexander C, Yurkowski D, Ferguson S, Belt S. Identifying variable sea ice carbon contributions to the Arctic ecosystem: A case study using highly branched isoprenoid lipid biomarkers in Cumberland Sound ringed seals. *Limnology and Oceanography* 59, 1581-1589 (2014). DOI: https://doi.org/10.4319/lo.2014.59.5.1581

14. Carlyle CG, *et al.* Spatial variation in carbon source use and trophic position of ringed seals across a latitudinal gradient of sea ice. *Ecological Indicators* 145, 109746 (2022). DOI: https://doi.org/10.1016/j.ecolind.2022.109746

15. Brown TA, Belt ST, Piepenburg D. Evidence for a pan-Arctic sea-ice diatom diet in *Strongylocentrotus* spp. *Polar Biology* 35, 1281-1287 (2012). DOI: https://doi.org/10.1007/s00300-012-1164-9

16. Brown TA, Galicia MP, Thiemann GW, Belt ST, Yurkowski DJ, Dyck MG. High contributions of sea ice derived carbon in polar bear (*Ursus maritimus*) tissue. *PLOS ONE* 13, e0191631 (2018). DOI: https://doi.org/10.1371/journal.pone.0191631
